# Risk assessment of heavy metals in coastal sediments of the Red Sea in Egypt

**DOI:** 10.1038/s41598-025-07135-x

**Published:** 2025-06-27

**Authors:** Mahmoud A. Dar, Amany G. Madkour, Ahmed R. Elgendy, Ghada Y. Zaghloul, AbdElMohsen S. ElDaba

**Affiliations:** National Institute of Oceanography and Fisheries, NIOF, Cairo, Egypt

**Keywords:** Sheltered zones, Heavy metals, Terrigenous and biogenic sediments, Contamination, Ecological risk, Biogeochemistry, Risk factors, Environmental sciences, Environmental impact

## Abstract

The Red Sea’s near-shore zones are considered nurseries and grazing grounds for the various economic fish species. To illustrate the relation between human health and seafloor sediments, the geological and geochemical properties of seafloor sediments were investigated in near-shore zones at Marsa Alam and Hurghada cities along the Red Sea. The obtained data illustrated that the sediment nature at Hurghada is primarily of biogenic origin, as indicated by the high carbonate contents; however, the sediment nature at Marsa Alam is attributed mainly to the terrigenous origin. Accordingly, the studied heavy metals at both localities showed different feeding sources; Marsa Alam sites showed high levels of Fe, Mn, Zn, Ni, and Cu attributed to terrigenous inputs; however, the high averages of Cd and Pb at Hurghada indicating influence from land-based and anthropogenic activities. The calculated risk assessment parameters and carcinogenic risk (ILCR) do not indicate any significant risk. Geochemically and as indicated by the statistical parameters: correlation coefficient, PCA, and Geo-accumulation (Igeo); Mn, Zn, Cu, and Ni were found to be mainly associated with Fe in the same source of accumulation and similar geochemical forms. However, the adsorption over sediment particles and/or assimilation inside the carbonate lattices are possible occurrences of Cd, Pb, and partially Ni. The calculated risk assessment parameters and carcinogenic risk (ILCR) do not indicate any significant risk to marine organisms and human consumption.

## Introduction

Marine sediments have a heterogeneous origin and exhibit significant variation in composition. Because of the differences in origin and formation conditions, sediments are classified as: lithogenous, biogenous, or authigenic. Lithogenous sediments are transported and dispersed into the sea as detrital particles from natural floods, smoothers, and coastal erosion due to coastal activities^1–3^. The biogenous sediments are formed by benthic organisms or from skeletal fragment accumulations, whereas, the authigenic sediments (hydrogenous) were precipitated from chemical solutions as new formations or as matrix materials inside or replacing the old formations^1^. Marine sediments in sheltered shallow areas are a mixture of the categories mentioned above and frequently act as functional repositories for different elements. Heavy metals are among these substances, occurring in marine environments in various forms, including suspended solids, dissolved ions, complexes, colloids, and sediment-bound solids^2,4^. Heavy metals have the potential to build up in aquatic systems’ organisms and move up the food chain. The presence of heavy metal pollution in seafloor sediments poses a threat to human health and the ecosystem^5^. Furthermore, because of these metals’ toxicity, bioaccumulation, persistence, and bio-magnifications in food chains, they pose a risk to human health and the ecological system^6^. Numerous disruptions and significant strains on the near-shore environment have resulted from the rapid and unregulated coastal activities along the Red Sea in recent decades^2,7^. Therefore, people as well as the near-shore fertile zones may be at risk due to the ongoing release of heavy metals into marine ecosystems^8^. In this regard, evaluating the ecological and health implications associated with heavy metals in marine sediments is crucial because of their significant influence on the marine environment and edible biota^9–11^.

To demonstrate the relationship between seafloor sediment and human health, this study will assess the effects of human activity, coastal development, and natural runoff on the sediment nature of the near-shore and sheltered zones off Hurghada and Marsa Alam, these zones are thought to be the most important grazing and nursery areas for commercial fish in the Red Sea. The assessment of potential ecological and health risks linked to heavy metals in seafloor sediments is another goal of this research.

## Materials and methods

### Environmental description

Marsa Alam’s sites (SI to SIV) include three semi-closed sheltered lagoons and a small embayment between them with a maximum depth of about 6 m in the near-shore zone of the Red Sea. These sites are home to dense seagrass beds, diverse coral reef communities, and a variety of benthos. The investigated sites at Hurghada (SV to SVIII) were located in the marine area off the National Institute of Oceanography and Fisheries (NIOF) and had varying ecological geomorphic features. These include crescent reefs, shallow lagoons, and diving sites with diverse benthic communities such as coral reef and coral patches, seagrass beds, and dense macro-algal blooms. The investigated sites at Marsa Alam and Hurghada were situated in front of two major Red Sea cities and were heavily impacted by temporary flash floods, high sedimentation rates subsurface seepage from domestic sewage, boats mooring safari boats, landfilling from land reclamation at the closest domestic villages, and the rejection of distillation plants in the tidal flat zones.

### Field works and laboratory treatments

**Fig. 1:**
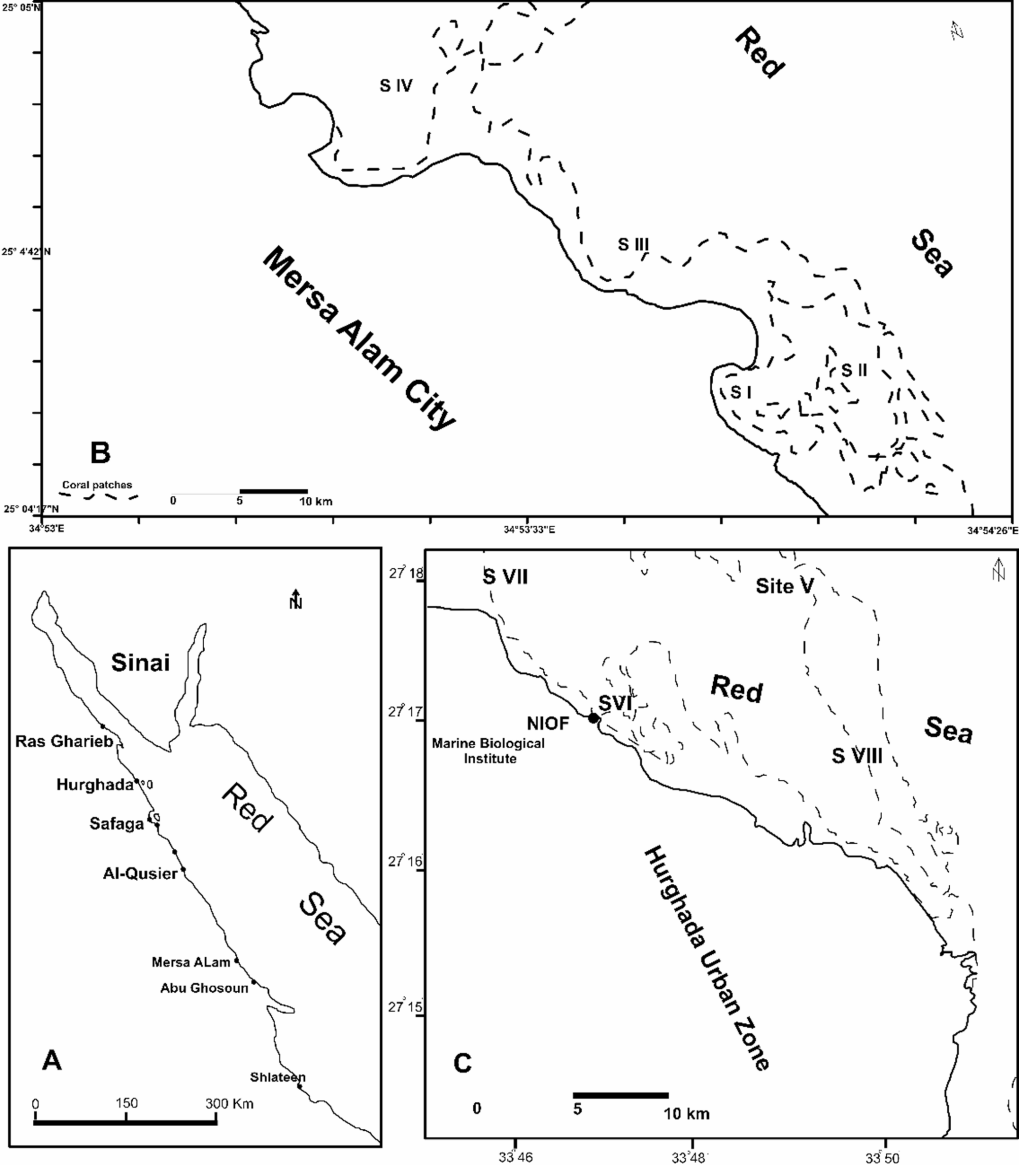
Location map shows the studied sites at Hurghada and Mersa Alam.

A total of nineteen sediment samples from Marsa Alam and twenty-five from Hurghada were collected from the near-shore zones of the Red Sea using a small boat and a grab sampler (Fig. 1). The collected samples were packed in polyethylene bags, then frozen and preserved in 4°C. At the laboratory, 100 g of the air-dried and disaggregated samples were subjected to grain size analyses each one phi (Ø) fractions according to^12^. The obtained fractions were categorised into three groups; the Coarse Sediment Group (CSG) involves (Ø-1 + Ø0), the Medium Sediment Group (MSG) involves (Ø-1 + Ø0) and the Fine Sediment Group (FSG) involves (Ø3 + Ø4 + <Ø4). The collected samples were prepared to be examined for geochemical characteristics by grinding about 5 g of the pre-homogenized samples using Corundum Mortar to less than 80 meshes (177 microns).

### Geochemical analyses

#### WCaCO_3_ and total organic matter (TOM%) determinations

WCaCO_3_ was determined by hydrolyzing one gram of each of the pre-grinded samples with 12% of glacial acetic acid overnight. The remaining insoluble residue after acid washing was dried, weighted then the carbonate percentage was calculated from the following Eq. (1):

1 $$\:\text{Carbonate\%=}\frac{\text{wt.of sample}\text{}-\text{}\text{wt.of residue}}{\text{wt.of sample}}{\times 100}$$

Determination of total organic matter percentage (TOM%) estimated by sequential ignition weight loss at 550 °C^13^ from the Eq. (2):

2 $$\:\text{T}\text{O}\text{M}\text{\%}=\frac{\text{w}\text{t}.\text{o}\text{f}\:\text{s}\text{a}\text{m}\text{p}\text{l}\text{e}\:-\:\text{w}\text{t}.\:\text{o}\text{f}\:\text{A}\text{s}\text{h}}{\text{w}\text{t}.\:\text{o}\text{f}\:\text{s}\text{a}\text{m}\text{p}\text{l}\text{e}\:}\times\:100$$

#### Leachable heavy metals determination

To ascertain the fractions of heavy metals that can be leached from bulk samples and fine fractions (Ø3, Ø4, Ø<4), 0.5 g of each sample was digested using a combination of Conc.nitric acid (HNO_3_) and Conc.perchloric acid (HClO_4_) until almost dry^14,15^. Using distilled water, the digested samples were diluted to 25 ml before filtering to remove any insoluble materials, then the leachable contents of; Fe, Mn, Zn, Cu, Pb, Ni, and Cd were determined in each of the filtrated samples using a flame atomic absorption spectrophotometer (AAS, GBC-932) at the National Institute of Oceanography and Fisheries in Hurghada, Red Sea, Egypt. The estimated results were given in µg/g. The obtained data were checked using certified Sigma-Aldrish reference material. The maximum accuracy is obtained by performing three replicates of each measurement with less than 3% deviation.

### Pollution assessment

Mathematical environmental indices are helpful instruments for the analysis and simplification of gathered data to assess the degree and the source (natural or man-made), accessibility, and toxicity of the anticipated environmental risk and PTE contamination^16,17^. To differentiate between the naturally occurring elements and their recently higher concentrations as a result of various impacts, the estimated results were interpreted concerning the averages of the natural background levels^15^.

#### Geo-accumulation (*I*_*geo*_), the contamination factor (C_*f*_) and the degree of contamination (C_deg_)

The geo-accumulation (I_geo_) and contamination factor (C_f_) indices were employed by^18,19^ to quantify the anthropogenic inputs of elements under investigation above their natural levels in marine sediments, Eqs. (3) and (4). To assess the sediment ecosystem quality of the sites under investigation^19,20^, computed integrated pollution indices, such as the degree of contamination (C_deg_) Eq. (5) in the event of multi-element contaminations.

3 $$I_{{geo}} = \log 2\frac{{C_{s} }}{{1.5~C_{b} }}$$

4 $${\text{C}}_{f} = ~\frac{{C_{s} }}{{C_{b} }}$$

C_s_ is the potential toxic element concentration (PTE) in the investigated metals and C_b_ is the background value. The severity values of (*I*_*geo*_) were classified into seven grades; practically unpolluted (*I*_*ge*o_≤ 0), unpolluted to moderately polluted (0 < *I*_*geo*_≤1), moderately polluted (1 < *I*_*geo*_≤2), moderately to highly polluted (2 < *I*_*geo*_ ≤3), highly polluted (3 < *I*_*geo*_≤4), highly to extremely high polluted (4 < *I*_*geo*_ ≤5) and extremely high polluted (*I*_*geo*_ >6).

5 $${\text{C}}_{{{\text{deg}}}} = \mathop \sum \nolimits_{{i = 1}}^{n} C_{f}$$

Whereas, n is the number of the examined PTEs. Based on the C_f_ values, the contamination degrees (C_deg_) were sub-divided into four class: low contamination (< 1), moderate contamination (1–3), considerable contamination (3–6), and very high contamination (> 6).

#### Potential ecological risk index (RI)

The potential ecological risk index (RI) was calculated according to Eq. (6) and it has been used to assess the ecological risk degree of heavy metals in aquatic sediments^21^ based on the individual ecological risk factor (Eri) that was suggested by^19^ to assess the individual ecological risk Eq. (7).

6 $${\text{RI}} = \mathop \sum \nolimits_{{i = 1}}^{n} Eri$$

Four classes of RI were obtained: low (< 150), moderate (150 ≤ RI ≤ 300), high (300 ≤ RI ≤ 600) and very high (RI ≥ 600).

7 $${\text{Eri}} = {\text{C}}_{{{\text{fi}}}} {\text{T}}_{{{\text{ri}}}}$$

Whereas, T_ri_ was the toxicological response factor for each toxic element^19^ (1, 5, 5, 5, and 30 for Zn, Pb, Cu, Ni, and Cd, respectively), and n is the number of investigated metals. The Eri was categorised into 5 categories; low (Eri < 40), moderate (40 ≤ Eri < 80), considerable (80 ≤ Eri < 160), high (160 ≤ Eri < 320) and very high (Eri > 320).

#### Cancer risk (ILCR) probability

The ILCR was used to determine the likelihood that exposure to heavy metal contamination in the marine environment would increase the risk of cancer^22^. Equation (8) defines the ILCR as the incremental likelihood that a person would get cancer throughout their lifetime due to exposure to a chemical or contaminants^23^.

8 $${\text{ILCR}} = {\text{CdI}} \times {\text{CSF}}$$

The Chronic Daily Intake (CdI) is measured in milligrams per kilogram per day and is absorbed through both oral and dermal contact. The risk created by a lifetime average of one mg/kg/day of carcinogenic chemicals is known as the cancer slope factor (CSF).

### Data treatment

Principal Component Analysis (PCA) is the most often used analysis tool for examining multidimensional datasets with quantitative variables using multivariate data analysis techniques. XLSTAT is a very useful tool for the analysing data. This software is an add-in tool for Excel that performs statistical and data analysis for forecasting, regression and quality control.

Golden Software Surfer Ver. 13.0 was used to plot the location map, and the relationships between the total trace metals were tested using the XLSTAT (2018) software, applying the correlation matrix, the principal component analysis (PCA) and the relation between elements and samples. Win Graph Prism Software Ver. 8.00 was used to plot the other figures.

## Results

### Grain size distribution

As shown in (Table 1), at Marsa Alam, Site SI showed the highest averages of CSG, Ø-1, Ø0, Ø1 and Ø4 (26.42, 8.18, 18.24, 16.87, and 24.42%, respectively), Site SIII recorded the highest MSG average (36.01%), however, Site SII has the highest averages of FSG, Ø2 and <Ø4 (58.62, 22.69 and (6.44%). Site SIV of Marsa Alam recorded the highest Ø3 average (38.68%). At Hurghada, Site SVII showed the highest averages of CSG and Ø0 (34.50 and 22.09%), and Ø-1 had the highest sub-equal averages at sites SVI and SVII (12.67% and 12.41% respectively). Site SVIII recorded the highest averages of MSG and Ø1 (45.18 and 25.99%), whereas, Ø2 showed nearly equally high averages at sites SVI and SVIII (19.37 and 19.19%). Site SVI at Hurghada showed the highest averages of FSG, Ø3, Ø4, and <Ø4 (49.71, 24.35, 19.66, and 5.70%, respectively).

**Table 1:** The averages of the sediment sizes distribution at the studied sites of Mersa Alam and hurghada.

Locality	Sites	Ø_−1_%	Ø_0_%	CSG%	Ø_1_%	Ø_2_%	MSG%	Ø_3_%	Ø_4_%	Ø_<4_%	FSG%
Mersa Alam	SI	8.18	18.24	26.42	16.87	12.05	28.92	17.45	24.42	2.79	44.66
	SII	2.11	4.33	6.44	12.04	22.69	34.73	33.37	18.8	6.44	58.62
	SIII	7.9	11.76	19.66	14.73	21.28	36.01	30.86	11.73	2.02	44.61
	SIV	8.08	7.66	15.75	12.9	20.24	33.14	38.68	11.06	1.31	51.06
Hurghada	SV	12.67	18.37	31.04	22.03	19.37	41.4	17.93	6.55	2.39	26.87
	SVI	5.92	10.87	16.79	16.02	17.38	33.4	24.35	19.66	5.7	49.71
	SVII	12.41	22.09	34.5	23.39	17.55	40.94	15.46	7.14	1.85	24.45
	SVIII	11.26	15.43	26.69	25.99	19.19	45.18	20.96	8.09	0.7	29.75

### Geochemical characteristics

Hurghada sites often exhibited the highest CO_3_% averages among all sites, indicating significant biological contributions. The highest average CO_3_% (84.73%) was reported at Site SVII in Hurghada (NIOF), while Site SI in Marsa Alam recorded the highest average CO_3_% (54.62%). Additionally, Site SVIII in Hurghada displayed the highest TOM average (4.70%), while Site SI in Marsa Alam had the greatest TOM average (3.55%) (Table 2).

**Table 2:** The average percentages of CO_3_% and TOM at the studied sites.

Sites	Mersa Alam		Hurghada		
	CO_3_%	TOM%	Sites	CO_3_%	TOM%
SI	54.62	3.16	SV	82.24	3.63
SII	33.98	1.98	SVI	79.07	3.40
SIII	32.34	3.04	SVII	84.73	2.92
SIV	52.15	3.55	SVIII	79.99	4.70

In the bulk sediments of Marsa Alam, Site SIII had the highest average concentrations of Fe, Zn, and Ni (30709.5, 57.40, and 84.88 µg/g, respectively), whereas Site SIV had the highest average concentrations of Mn (327.50 µg/g). The highest averages of Pb and Cd in the bulk sediments were at Site SI (27.16 and 1.36 µg/g), while the highest Cu average was at Site SII (21.61 µg/g). In Ø3, Site SII had the highest averages of Mn and Cu (292.50 and 15.24 µg/g), but Site SIII had the highest averages of Fe, Zn and Ni (31901.83, 47.26 and 82.51 µg/g, respectively). The optimum averages of Pb and Cd in Ø3 (23.44 and 5.76 µg/g, respectively) were found in Sites SIV and SI. The high averages of Fe, Zn, Ni, and Pb were at Site SIII in Ø4 (30415.93, 44.46, 65.68, and 16.00 µg/g, respectively). Site SII recorded the greatest averages of Mn and Cu (256.10 and 11.56 µg/g) in Ø4, while Site SI recorded the highest average of Cd (1.73 µg/g). Site SII had the highest averages of Fe and Mn (20839.00, 211.90, and 9.42 µg/g) in the finest fraction (Ø<4). Additionally, Site SIV had the highest averages of Zn and Pb (44.11 and 19.34 µg/g), whereas Sites SIII and SI had the highest averages of Cu and Cd (16.42 and 1.80 µg/g), respectively in Ø<4 (Table 3).

**Table 3:** The average concentrations of heavy metals in the bulk sediments and the finest fractions (Ø_3_, Ø_4_ and Ø_<4_) in (µg/g) at The different sites of Mersa Alam and hurghada.

Locality	Sed. Size	Sites	Fe	Mn	Zn	Cu	Ni	Pb	Cd
Mersa Alam	Bulk	SI	12542.40	184.60	33.25	10.63	37.12	27.16	1.36
		SII	28982.69	325.00	55.09	21.61	74.92	7.54	0.48
		SIII	30709.53	282.60	57.40	19.57	84.88	5.83	0.34
		SIV	23286.12	327.50	41.02	13.22	60.38	13.16	0.65
	Ø_3_	SI	11923.40	171.50	32.02	7.16	44.14	20.61	5.76
		SII	29468.83	292.50	46.34	15.24	77.36	13.83	1.53
		SIII	31901.83	274.30	47.26	14.73	82.51	15.39	1.46
		SIV	24047.92	273.75	36.75	11.94	68.72	23.44	1.70
	Ø_4_	SI	10378.40	186.41	30.33	6.29	40.19	13.84	1.73
		SII	26410.76	256.10	37.14	11.56	60.79	14.80	1.41
		SIII	30415.93	222.60	44.46	9.01	65.68	16.00	1.09
		SIV	25124.38	198.88	36.74	9.91	59.27	15.99	1.53
	Ø_<4_	SI	14303.00	200.23	37.04	8.92	56.20	14.27	1.80
		SII	20839.00	211.90	36.43	12.71	50.05	17.57	1.72
		SIII	17525.49	139.30	31.56	16.42	42.29	16.66	1.22
		SIV	19393.68	153.50	44.11	8.44	46.65	19.34	1.47
Hurghada	Bulk	SV	297.00	9.36	13.08	2.52	16.71	25.65	1.19
		SVI	1080.33	34.43	17.47	3.96	16.24	23.93	0.18
		SVII	244.00	7.57	10.97	2.89	20.08	23.13	1.26
		SVIII	537.33	21.48	12.49	7.79	19.83	18.92	0.52
	Ø_3_	SV	222.43	7.81	9.64	2.11	21.85	35.00	4.90
		SVI	1403.33	33.35	13.26	7.21	19.96	29.93	11.32
		SVII	331.11	10.19	9.77	2.32	17.96	35.80	3.30
		SVIII	564.83	22.10	9.54	2.15	16.63	33.15	3.60
	Ø_4_	SV	347.86	10.22	12.20	2.34	21.19	35.99	14.20
		SVI	1568.00	44.50	12.63	3.13	20.32	26.68	11.07
		SVII	407.44	12.99	10.01	2.62	18.32	34.36	4.11
		SVIII	766.17	33.16	10.34	2.20	15.30	28.18	3.35
	Ø_<4_	SV	472.43	12.79	10.99	1.22	23.36	37.59	4.23
		SVI	1925.33	59.25	14.26	2.40	19.45	28.07	3.82
		SVII	548.56	16.56	10.37	2.01	14.26	30.11	3.32
		SVIII	960.50	33.54	9.66	4.10	12.96	19.36	2.38
Red Sea Background Values^25^			3000	116	24	17.6	16	3	0.4
Upper continental crust^27^			35,000	600	71	25	20	20	< 1.00
Bulk continental Crust^26,27^			70,700	1400	80	75	105	8	< 1.00

At Hurghada, the bulk sediments showed the highest averages of Fe, Mn, and Zn (1080.33, 34.43, and 17.47 µg/g) in Site SVI, while Site SVII had the highest averages of Ni and Cd (20.08 and 1.26 µg/g). Site SVIII exhibited the greatest average of Cu (7.79 µg/g), while Site SV recorded the highest Pb (25.65 µg/g). The optimum averages of Fe, Mn, Zn, Cu, and Cd (1403.33, 33.35, 13.26, 7.21, and 11.32 µg/g, respectively) in Ø3 were found at Site SVI. The bulk sediments from Site SVI exhibited the highest averages of Fe, Mn, and Zn (1080.33, 34.43, and 17.47 µg/g), while Site SVII had the highest averages of Ni, and Cd (20.08, and 1.26 µg/g). Site SVIII exhibited the greatest average of Cu (7.79 µg/g), however, Site SV recorded the highest Pb (25.65 µg/g). In Ø3, the greatest averages of Fe, Mn, Zn, Cu, and Cd (1403.33, 33.35, 13.26, 7.21, and 11.32 µg/g, respectively) were found at Site SVI, Site SV recorded the highest Ni (21.85 µg/g) and Site SVII showed the highest Pb average (35.80 µg/g). In Ø4, the highest averages of Ni, Pb, and Cd (21.19, 35.99, and 14.20 µg/g) were found at Site SV, while the highest averages of Fe, Mn, Zn, and Cu (1568.00, 44.50, 12.63, and 3.13 µg/g) were found at Site SVI. The finest fraction (<Ø4) showed the highest averages of Fe, Mn, and Zn (1925.33, 59.25, and 14.26 µg/g) at Site SVI, Site SV recorded the highest Cd, Pb, and Ni (4.23, 37.59 and 23.36 µg/g), however, Site SVIII exhibited the highest average of Cu (4.10 µg/g). Compared to the Red Sea background levels of heavy metals reported by^24^, the Mersa Allam sites exhibited elevated concentrations of Fe, Mn, Zn, Ni, Pb, and Cd, but a lower level of Cu. Conversely, the Hurghada sites demonstrated reduced concentrations of Fe, Mn, Zn, and Cu, whereas the levels of Ni, Pb, and Cd were notably higher. Furthermore, the data recorded for the investigated sites at both localities revealed significantly lower concentrations of Fe, Mn, Zn, and Cu in comparison to the average values in the Upper Continental Crust and Bulk Continental Crust as reported by^27^. However, Ni concentrations at Mersa Allam were relatively high, while at Hurghada, they fell within the range of the Upper Continental Crust, though in both locations, Ni values remained below the average for the Bulk Continental Crust. Notably, Pb and Cd concentrations at both sites exceeded the average values reported for both the Upper Continental Crust and Bulk Continental Crust (Table 3).

## Discussion

### Sediment nature and distribution

The sheltered areas of closed and semi-closed lagoons in the near-shore zones act as nurseries for fish larvae and various benthic organisms, providing food and protection against natural predators. At the same time, these ponds act as traps for the different sediment types, especially the fine-grained sizes that may load considerable quantities of heavy metals. Also, the slack conditions and the slightly limited water exchange in sheltered areas due to the ebb and tide phenomena (semi-diurnal tide) led to continuous changes between oxic and anoxic conditions in the sediment layer. The investigated sites had heterogeneous seafloor sediment layers (lithogenous, biogenous, and authigenic) resulting from terrestrial flash floods that threw large amounts of terrigenous sediments and the biological productions in the form of coral reef fragments and other skeletal benthos as well as the geochemical changes. Consequently, the different sites of Marsa Alam receive terrestrial inputs from the adjacent wadis much more than Hurghada Sites. In the sheltered and isolated locations, the bottom sediments retained most of the entering terrestrial heavy metals^28^. Simultaneously, the distribution of the large - sized sediments of the sheltered zones was explained by the waves winnowing action and the periodical longshore currents that disperse the fine constituents (2). At the same time, the biologically generated coral skeletons, coralline algae, foraminiferal tests, and other carbonate minerals are crucial for understanding the dynamics of the sediment^29^.

### **Geochemical characteristics of the surface sediments**

#### Carbonates and TOM contents

The high percentages of CGS and CO_3_% at Hurghada are attributed to three main factors: first, the high biological production of carbonate fragments from various benthos, including coral reefs, coralline algae, echinoderms, mollusks, and foraminiferal tests; second, Hurghada sites provide greater protection against current and winnowing waves than Marsa Alam; and the third, the significantly lower terrestrial income from flash floods^30^ defines different sources of biological production in the Red Sea sediments; peloids, coated grains, ooids, and grapestones^31^ claimed that a variety of creatures were adding to the coral reef’s framework lattice, including mollusks, bryozoans, sponges, echinoderms, brachiopods, coralline algae, Halimeda, and foraminifera.

The Total organic matter percentage (TOM%) showed slight variation between Hurghada and Marsa Alam, but there were no appreciable signs of anthropogenic activity or terrestrial runoff. In general, the presence of TOM within the sheltered areas is indicative of biological processes that occur naturally, such as the restoration of live benthic fauna and flora blooming^32^ linked the recorded TOM% in the near-shore sediments to the local hydrodynamics, algal and seagrass flourishing, the terrigenous inputs, and domestic wastewater seepage.

#### Heavy metal contents in superficial sediments

The remobilization of sediment-associated metals can occur according to natural events, such as tidal movement and storms, or due to human activities. Ref.^33^ indicated that the presence, distribution, and dominance of any metal phase in the inshore sediments are influenced by the cation exchange capacity, mineral composition, particle size, and oceanographic circumstances. The availability of heavy metals in sediments allows aquatic organisms to consume them^34^, which is taken into account while determining health risks. When consumed, some of the accumulated heavy metals in the edible portions of marine organisms may enter the human body^11,34^ documented that the uptake of heavy metals in the edible portions of marine organisms occurs mainly from water, food, and sediments.

**Fig. 2:**
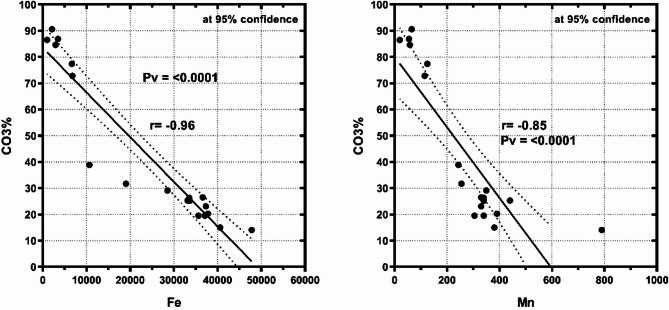
Significant negative correlations between CO_3_% and Fe (*r* = −0.96, *p* < 0.0001) and Mn (*r* = −0.85, *p* < 0.0001) in the bulk sediments of Mersa Alam.

**Fig. 3:**
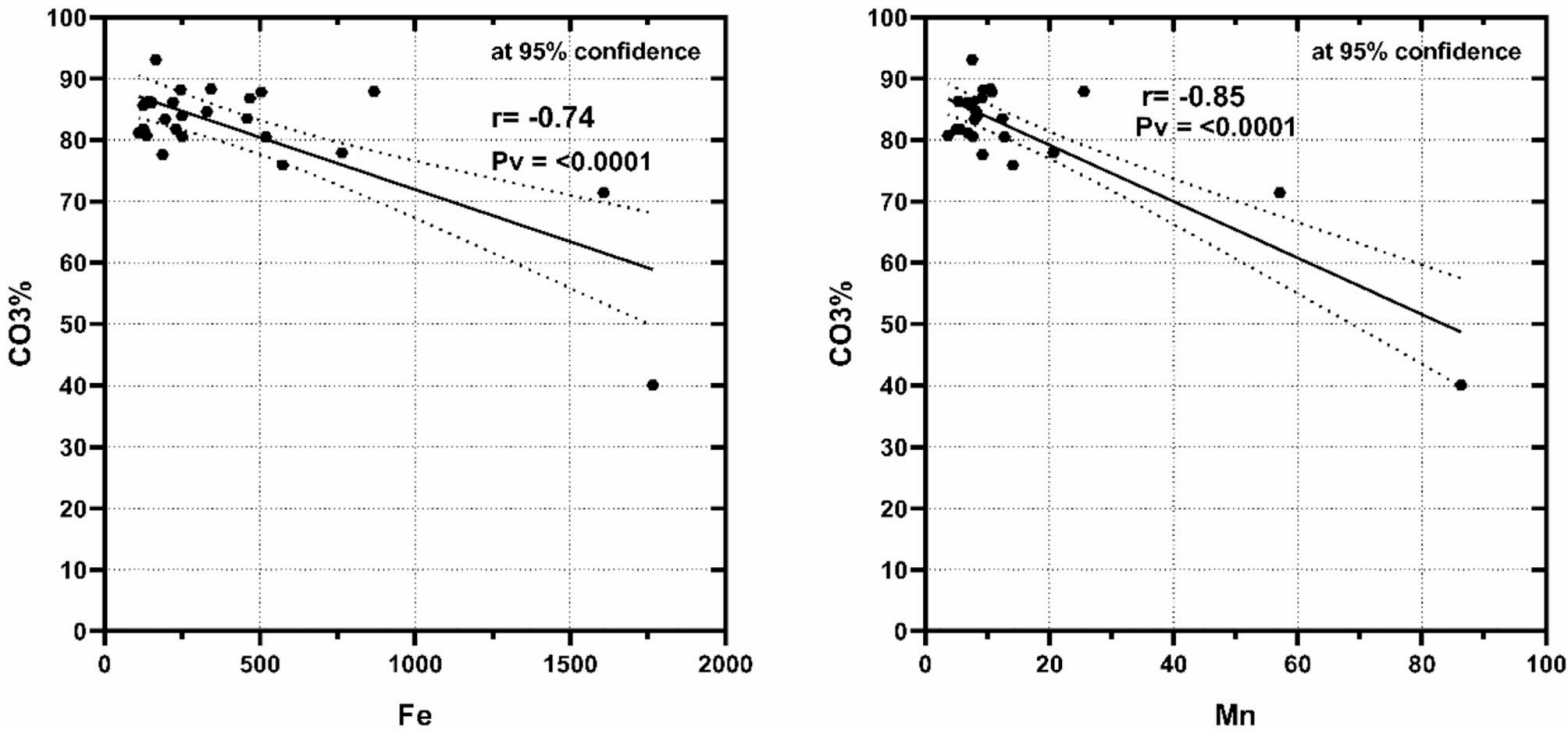
The negative correlations between CO_3_% and Fe (*r* = −0.74, *p* < 0.0001) and Mn (*r* = −0.85, *p* < 0.0001) in the bulk sediments of Hurghada.

By comparing the bulk sediments and the studied fine fractions (Ø3, Ø4, Ø<4) at the various Marsa Alam with Hurghada sites, the high terrestrial income from flash floods in Marsa Alam may be the reason for the greatest percentages of Fe, Mn, Zn, Cu, and Ni in the locality, where, Flash floods in the Red Sea region, particularly in Hurghada and Mersa Allam, serve as natural contributors to the heavy metal loads in these areas. These flash floods primarily transport sediments resulting from the natural erosion of surrounding soil and rocks, which are often rich in heavy metals such as iron, manganese, nickel, lead, and cadmium. Additionally, flash floods may carry salts and minerals deposited in nearshore zones due to atmospheric factors such as dust and airborne particles. The composition of these sediment loads largely depends on local geology, the flash flood’s pathway, and its intensity. Consequently, flash floods play a significant role in influencing the dynamics of heavy metal distribution along the coastal areas of the Red Sea. Conversely, the increased biological output was the cause of the decreasing Fe and Mn percentages in the Hurghada sediments. The fact that Mn and Fe were found in Marsa Alam demonstrated how these metals enter the coastal environment through terrestrial runoff, whereas they are linked to anthropogenic fine and particle sediments in Hurghada. These investigations were validated by the strong negative correlations of Fe and Mn with CO_3_% at Marsa Alam (−0.96 and − 0.85) (Fig. 2) and Hurghada (−0.74 and − 0.86) (Fig. 3). According to^2^, Fe and Mn are derived from the same terrestrial sources, are similar in nature and accumulation patterns, and have been associated in the forms of oxide, oxyhydroxide, and sulfide.

**Fig. 4:**
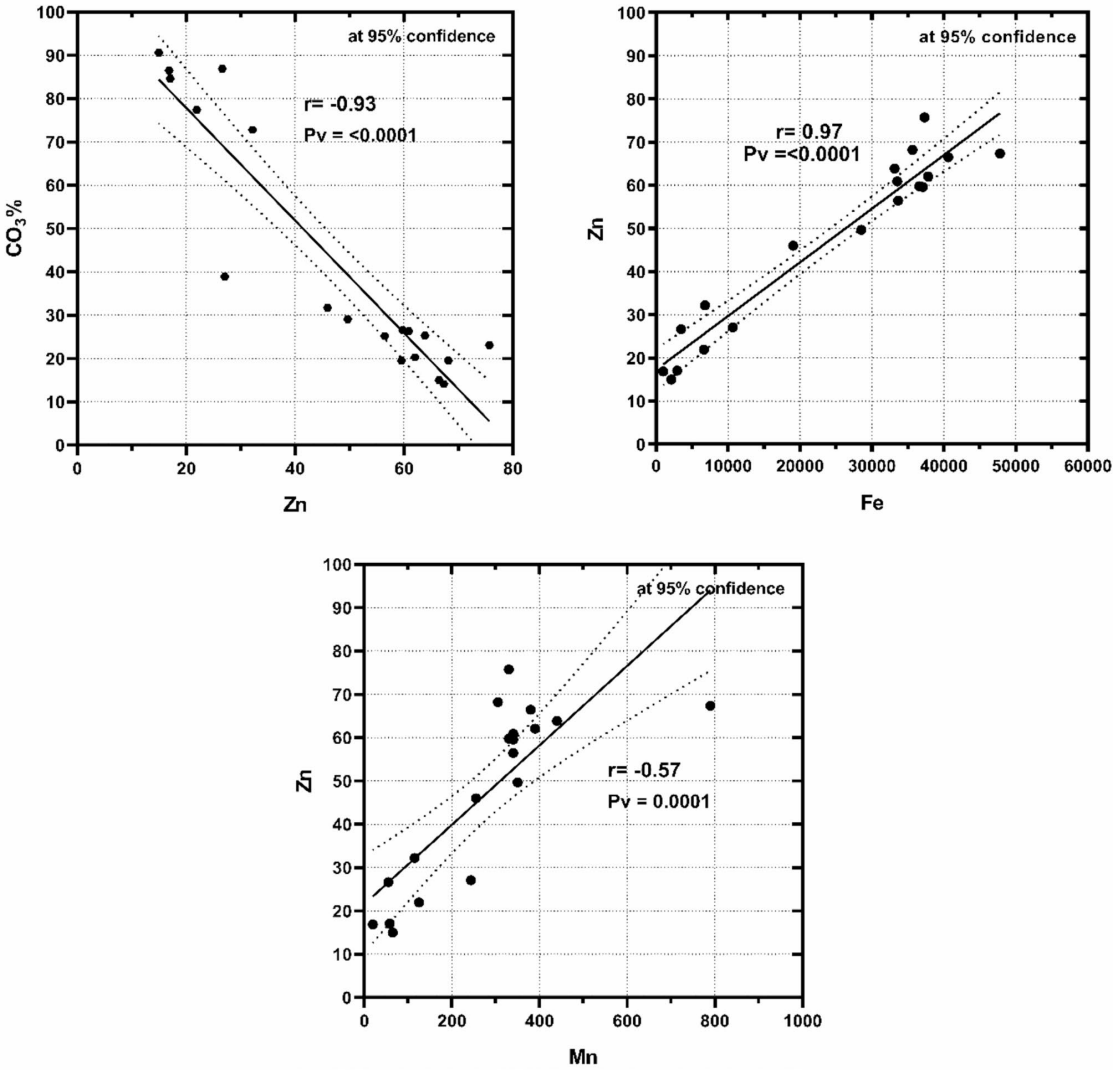
Inverse correlation of Zn with CO_3_%, and positive correlations with Fe and Mn in bulk sediments of Mersa Alam.

Zinc recorded high contents in the bulk sediments and the finest fractions at the different sites of Marsa Alam compared with those at Hurghada. This situation suggested that zinc in the sediments of the analyzed regions originates from a combination of sources, including terrestrial contributions, biogenic activities, and suspended particulate matter. Biogenic activity of Microorganisms critically governs zinc’s distribution in sediment. Microorganisms mediate its cycling through direct accumulation (biosorption, intracellular precipitation) and indirect speciation changes driven by biomineralization, redox shifts, and organic complexation. The analysis identifies two primary mechanisms of zinc accumulation in marine sediments: one is biological, involving the incorporation of ionic zinc into the carbonate structural lattice of marine benthic organisms, and the other is anthropogenic or naturally derived, linked to the association of zinc with other metals from lithogenic sources and terrestrial runoff^35–37^. The substantial strong negative correlation with CO_3_% and the significant strong positive correlation with Fe and a fair correlation positive with Mn (Fig. 4) suggested that the essential source of Zn in Marsa Alam was the terrestrial runoff. At Hurghada, Zn showed positive correlations with Fe and Mn (*r* = 0.62 and 0.52, respectively) in the bulk sediments and a weak negative correlation with CO_3_% (*r* = −0.32) indicating terrestrial sources of Zn mostly from suspended sediments^2,38^. suggested that the occurrence, migration capacity, and mobility of Zn were different according to the sediment type and size, seawater mixing, and oxic-anoxic conditions^39,40^. reported that Zn was related to Fe and Mn, particularly in the fine particle sediments.

**Fig. 5:**
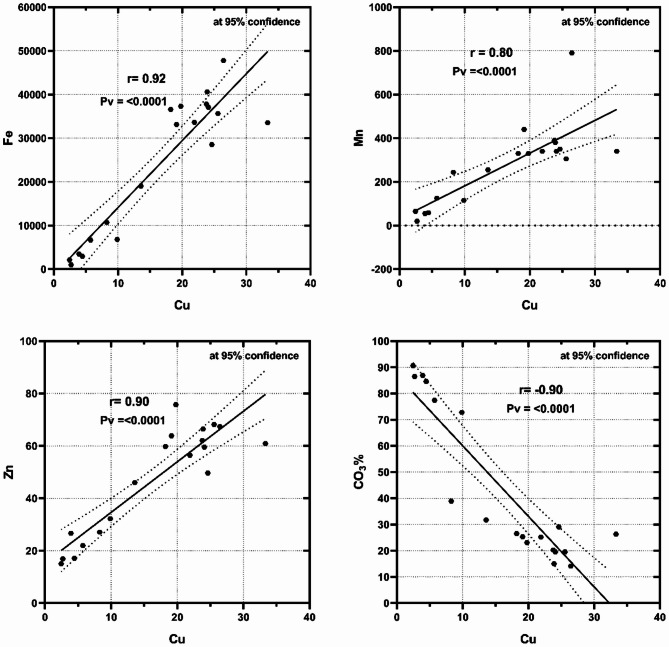
Cu shows positive correlations with Fe, Mn, and Zn, but an inverse correlation with CO_3_% in the bulk sediments of Mersa Alam.

Copper (Cu) is important for biological activities because it is necessary as a co-factor for many biochemical reactions, but when its contents exceed the natural levels, it becomes toxic to organisms and can cause damage by breaking DNA strands, which can lead to carcinogenesis. Anxiety, vomiting, diarrhea, hemolysis, liver necrosis, hematuria, proteinuria, hypotension, tachycardia, convulsions, and coma can all result from an increase in Cu concentration in human bodies^41,42^. The recorded values of Cu at both locations are significantly lower compared to those recorded by numerous authors in the Red Sea sediments^9,31,43^. The recorded Cu at the studied localities is attributed to the terrestrial runoff much more than the accumulation from biological or biochemical processes. According to^40,44^, Cu prefers to accumulate with organic compounds as sulfide and/or as adsorbed metal over other metal forms. It also exhibits a stronger interaction with carbonates, exchangeable phases, and Fe/Mn oxides/hydroxides. These investigations were supported by the strong to significant strong positive correlation with Fe, Mn, and Zn and a significantly strong negative correlation with CO_3_% in the bulk sediments at Marsa Alam (Fig. 5). In contrast, at Hurghada, Cu showed no correlation with any other metals, pointing to an unclear source of accumulation.

**Fig. 6:**
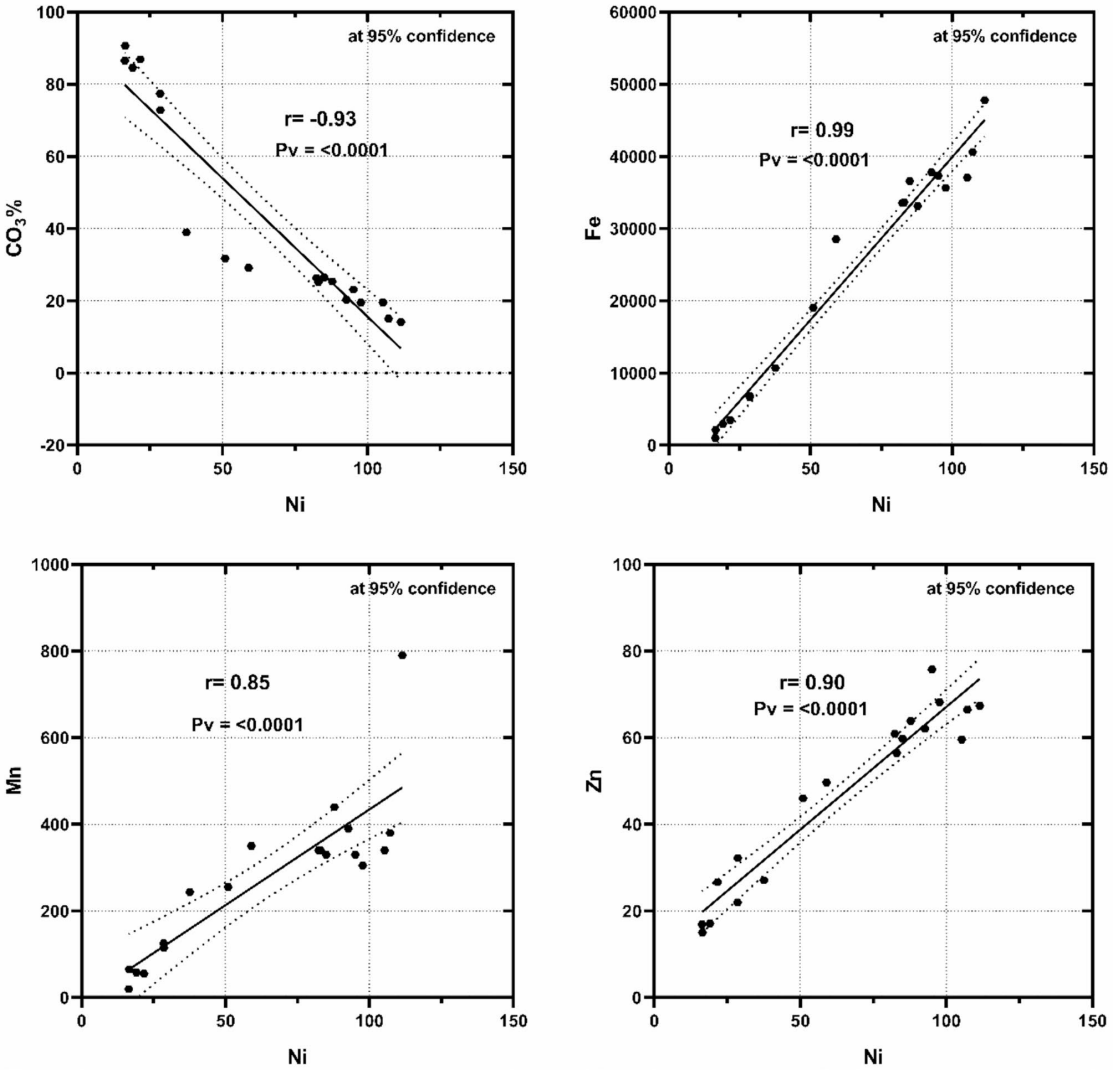
Ni shows strong negative correlation with CO_3_% and positive correlations with Fe, Mn, Zn, and Cu (*r* = 0.90) in the bulk sediments of Mersa Alam, suggesting accumulation from terrigenous sources rather than association with carbonates.

Because of its physiological and chemical characteristics, nickel (Ni) is one of the carcinogenic metals that contaminate the marine environment and then build up in the human body, endangering seafood consumers^11^. Exposure to nickel has been linked to malignancies of the lung, nose, and sinus tissues that exhibit carcinogenesis^45^. The bulk sediments and fine fractions (Ø3, Ø4, Ø<4) of the Marsa Alam exhibited significantly higher Ni content than the reported values at Hurghada, indicating a terrestrial source of accumulation^46,47^. stated that marine organisms may be hazardously affected by toxicity resulting from the elevated Ni levels introduced to marine ecosystems, both naturally and/or anthropogenically. Statistically, the correlation coefficient relationships provided support for the obtained suggestions. At Marsa Alam, Ni exhibited strong negative correlations with CO_3_% and positive correlations with Fe, Mn, Zn (Fig. 6), and Cu (*r* = 0.90), suggesting the accumulations from the same terrigenous sources neither with carbonates. However, at Hurghada, Ni misses such correlations with the terrigenous and organic sources indicating to anthropogenic source of accumulation or adsorption over sediment particles.

**Fig. 7:**
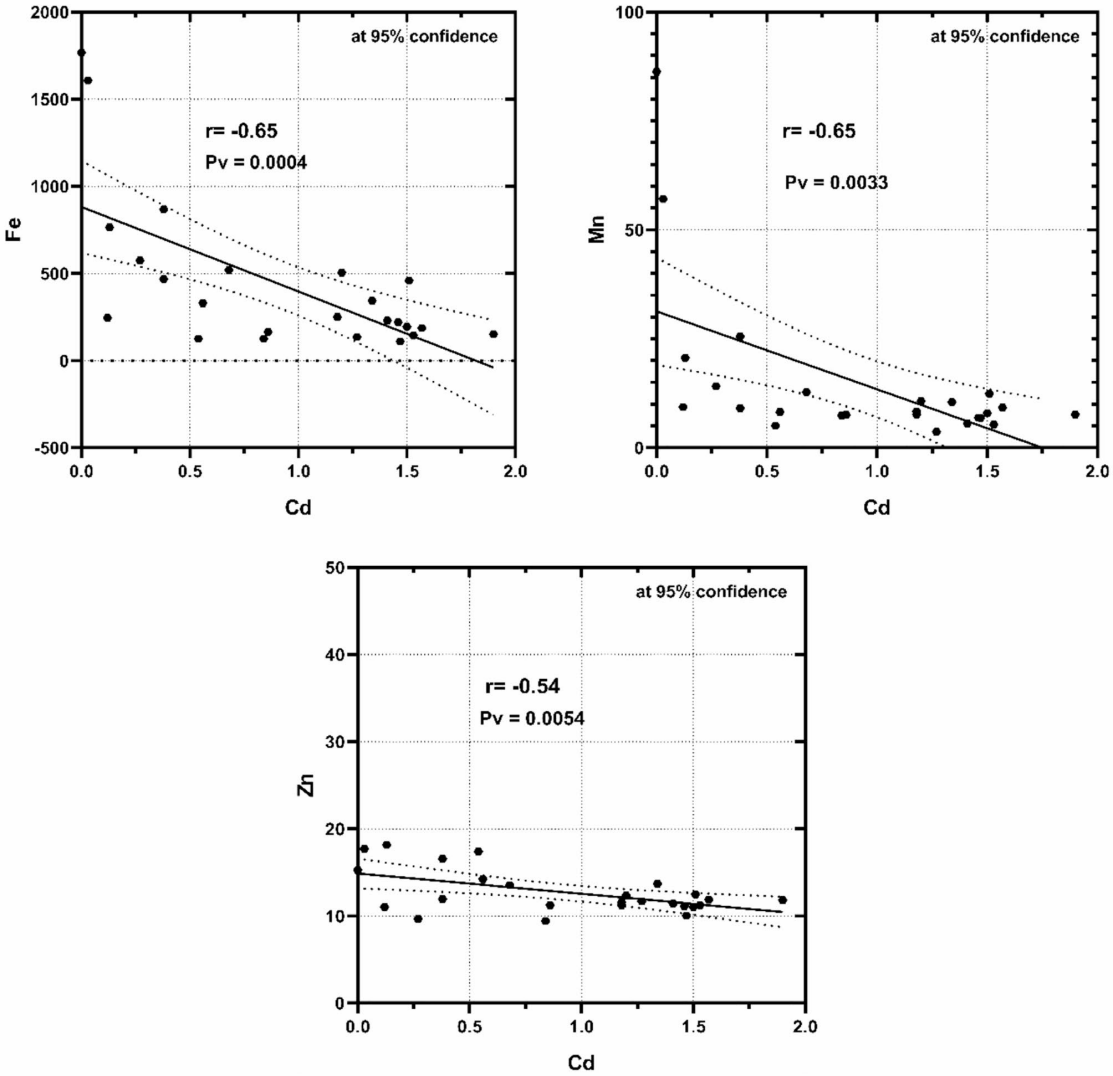
Cd exhibited a negative correlation with the terrestrial source metals; Fe, Mn, and Zn in the bulk sediments at Hurghada.

Cadmium (Cd) is rarely found in the marine environment, whereas it comes mostly from industrial and agricultural wastes. The IARC classified cadmium as a carcinogenic metal in class 1^48^. Long-term exposure to Cd may cause harmful effects on the different human organs; breast, esophagus, stomach, intestines, prostate, lungs and testes^49^. Cd showed abruptly high values in the different sediment sizes at the different sites of Hurghada compared with Marsa Alam, which might be attributed to the anthropogenic activities as; motor outboard, oil spill and fishing activities, reclamation activities, in addition to the subsurface wastewater seepage. The Cd contents at the two localities exhibited a negative correlation with the terrestrial source metals; Fe, Mn, and Zn (Fig. 7) at Hurghada, and Fe (*r*=-0.87), Mn (*r*=-0.79), Zn (*r*=−0.83), Cu (*r*=-0.81) at Marsa Alam. The strong positive correlation (*r* = 0.90) with CO_3_% at Marsa Alam indicated the accumulation from ionic form associated with carbonates (Table 4).

**Table 4:** The correlation coefficient relationships between different geochemical components of the studied sediments at Mersa Alam and hurghada.

		Fe	Mn	Zn	Cu	Ni	Pb	Cd	CO_3_%	TOM%	
Mersa Alam	Fe	1.00	0.95	0.62	0.14	-0.39	-0.62	-0.65	-0.74	-0.11	Hurghada
	Mn	0.89	1.00	0.52	0.19	-0.36	-0.71	-0.56	-0.86	-0.22	
	Zn	0.97	0.81	1.00	0.08	-0.32	-0.07	-0.54	-0.32	-0.24	
	Cu	0.92	0.80	0.90	1.00	0.19	-0.29	-0.07	-0.03	0.01	
	Ni	0.99	0.85	0.96	0.90	1.00	0.10	0.15	0.44	0.05	
	Pb	-0.93	-0.74	-0.91	-0.88	-0.94	1.00	0.39	0.65	0.12	
	Cd	-0.87	-0.79	-0.83	-0.81	-0.85	0.84	1.00	0.40	-0.17	
	CO_3_%	-0.96	-0.85	-0.93	-0.90	-0.93	0.86	0.92	1.00	0.14	
	TOM%	-0.57	-0.48	-0.57	-0.61	-0.57	0.52	0.42	0.56	1.00	

Lead (Pb) is one of the hazardous heavy metals that pose a serious risk to human health^11,50^. proposed that Pb can be introduced into the human body through contaminated foods and due to its chemical and physiological characteristics; it poses a health risk for seafood consumers, especially those in the daily diet. The recorded values of Pb at Hurghada are slightly higher than those at Marsa Alam in the bulk and different fraction sediments. Pb is mostly introduced to the marine environment essentially through land-based and human activities.

**Fig. 8:**
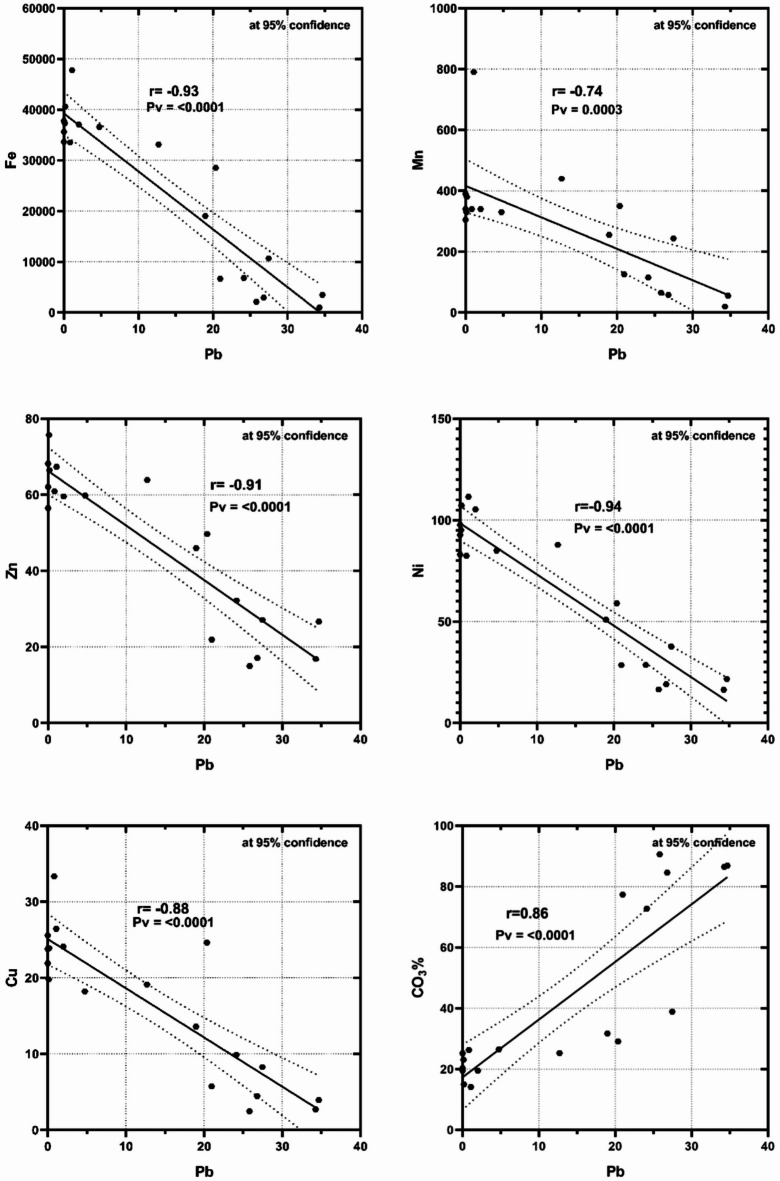
Negative correlations of Pb with the terrestrial source elements; Fe, Mn, Zn, Ni and Cu, and positive associations with CO_3_% at Marsa Alam, suggesting terrigenous input and the possibility of an independent anthropogenic source.

As in the Cd case, Pb exhibited negative correlations with the terrestrial source elements; Fe, Mn, Zn, Ni and Cu, and positive with CO_3_% at Marsa Alam (Fig. 8) suggesting the possibility of an anthropogenic independent source. Additionally, Pb showed negative correlations with Fe and Mn (*r*= −0.62, −0.71) at Hurghada confirming partially the same anthropogenic attitude. The recorded positive correlation of Pb with CO_3_% at Hurghada (*r* = 0.65) confirmed the accumulation of ionic forms associated with carbonates^31^. Conjectured that the elevated Pb concentration in the seafloor sediment of Hurghada could be attributed to Pb produced from unintentional oil spills and fuel seepage from mooring boats and ship maintenance. Industrial and land-based activities such as ship repair and maintenance, land reclamation, and other human activities are to blame for the reported Pb contents in the bottom sediments^11^.

### The interrelations between the different variables

**Fig. 9:**
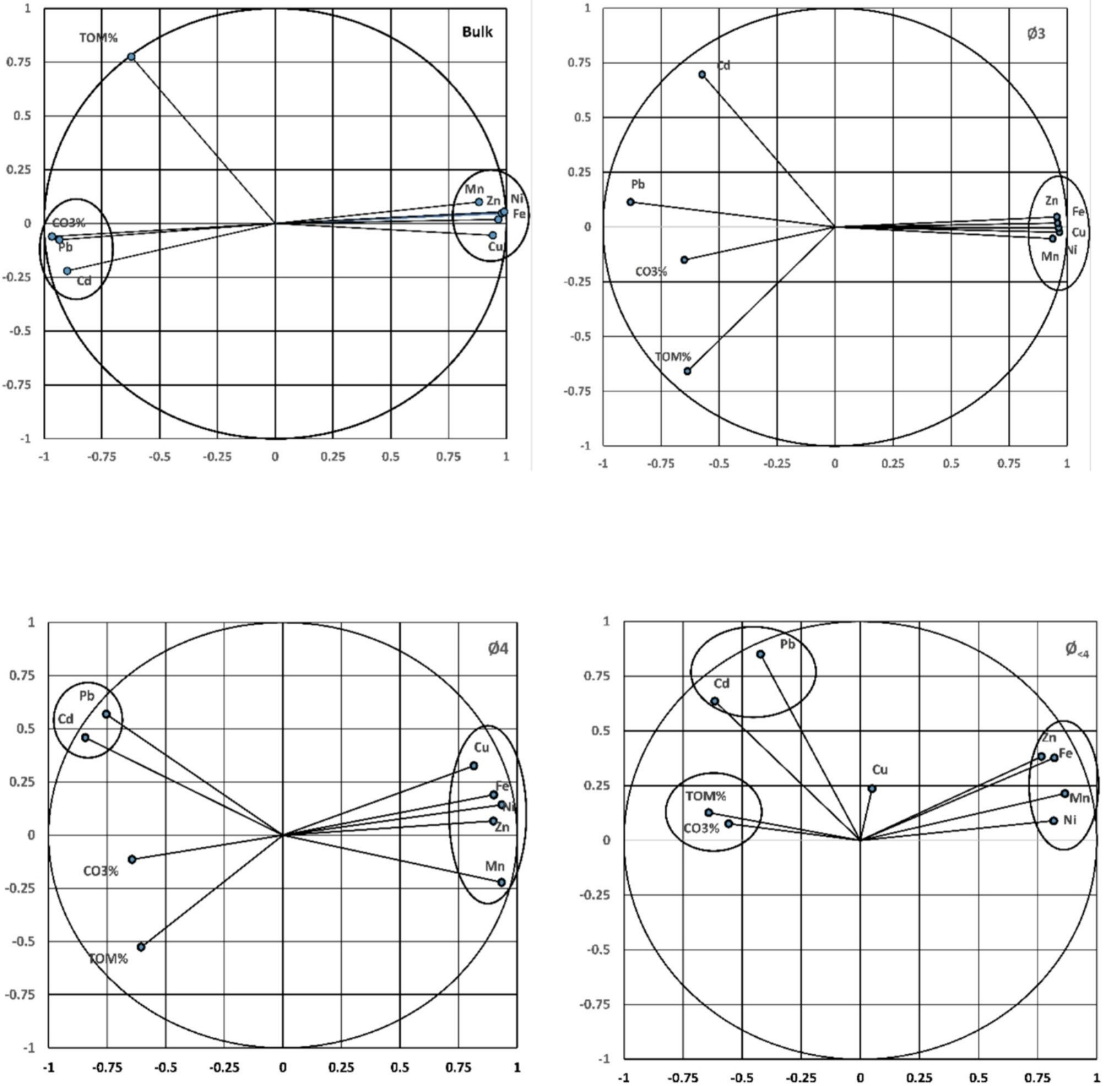
PCA bi-plots for Mersa Alam showing heavy metal associations with Fe, TOM, and CO₃% in both bulk and fine sediment fractions.

In this study, the estimated figures of PCA were applied to elucidate the interrelation between the sediment fractions, CO_3_%, and TOM%, to investigate the essential metal carrier and the potential sources of heavy metals. At Marsa Alam; Mn, Ni, Zn, and Cu were sequestered closely to Fe in the bulk sediments, and the fine fractions (Ø3, Ø4, Ø<4) with multivariate variances of; 91.70% in the bulk, 82.98% in Ø3, 79.20% in Ø4 and 61.22% in Ø<4 respectively, suggesting that they originated from the same chemical phase and source. However, in the bulk sediments and Ø3; Pb and Cd were sequestered with CO_3_%, suggesting that both metals are associated with carbonates in the structural lattice. In Ø4 and Ø<4, Pb and Cd were sequestered together, suggesting that there is another source of accumulation, mostly land-based and/or human activities (Fig. 9).

**Fig. 10:**
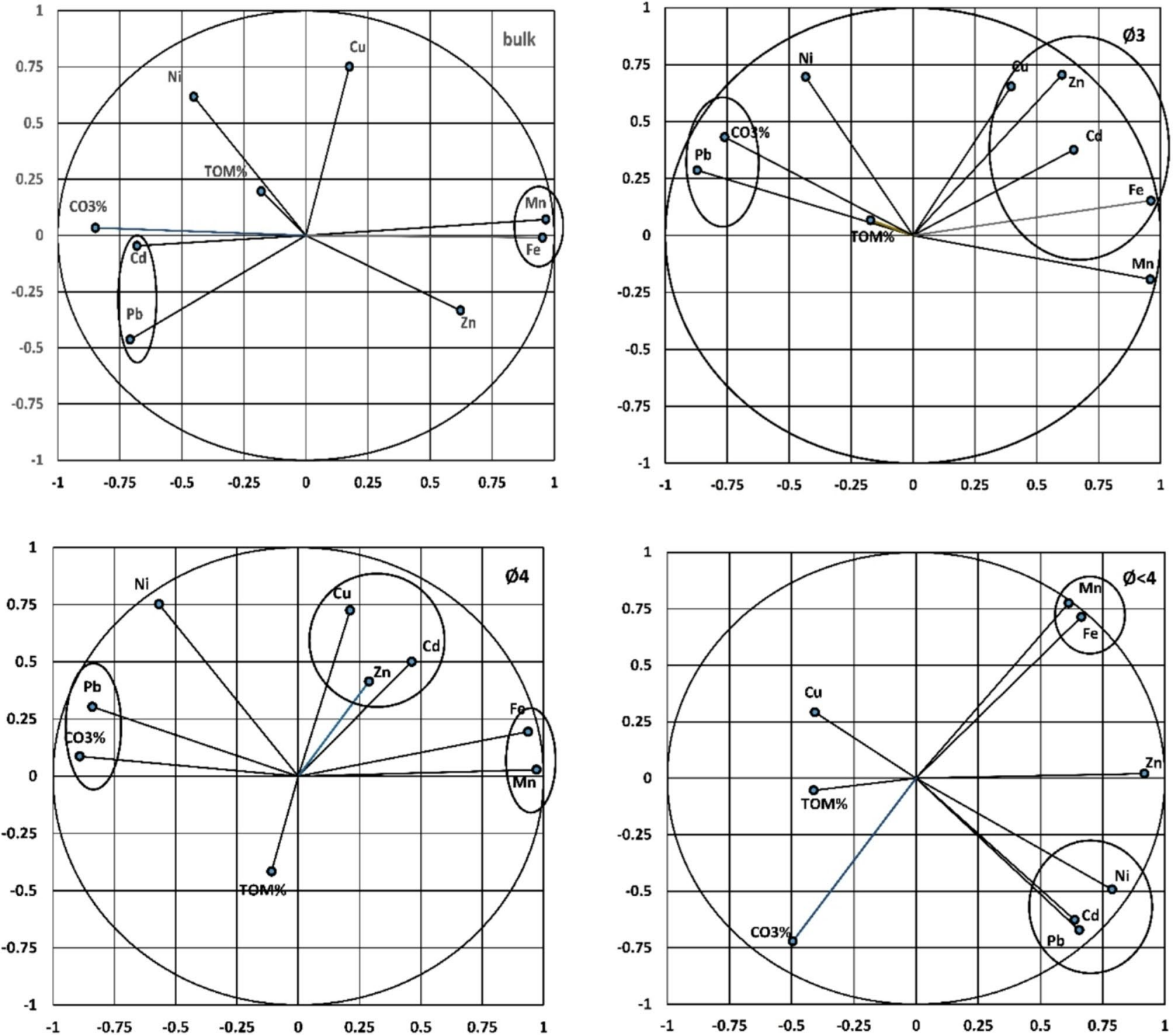
PCA bi-plot variables showing heavy metal tendencies toward Fe, TOM and CO_3_% in the bulk and fine fraction sediments at Hurghada.

At Hurghada, the multivariate variances of the studied components and metals showed significant variations, indicating multiple sources of metal occurrences. The multivariate sequesters were; 61.15% in the bulk sediments, 69.16% in Ø3, 64.71% in Ø4 and 72.29% in Ø<4 respectively. Fe and Mn are close to each other in one multivariate sequester and partially Cu and Zn suggesting that these elements have the same source and phase of occurrence, however, Ni seems to be carried by TOM mostly in the form of sulfide, meanwhile, Cd and Pb have tend CO_3_% as carbonate structures. In Ø3 and Ø4, Cd was sequestered with Mn, Zn, and Cu in association with Fe in one sequester attributing the accumulation to the landfilling in the sheltered zones, however, Pb and Ni were associated with CO_3_% in the carbonate structural lattice. The finest fraction (Ø<4) revealed all metals together in a single sequester, indicating the same source and geochemical form, except Cu which was clustered independently (Fig. 10).

### Geo-accumulation (*I*_*geo*_), the contamination factor (C_*f*_) and the degree of contamination (C_deg_)

As shown in (Table 5), the geo-accumulation index (I_geo_) showed negative signs of pollution for all of the investigated metals except Cd at both localities. The severity of (I_geo_) of Cd at Marsa Alam fluctuated between unpolluted to moderately polluted (0 < I_geo_ ≤1) bulk sediments to moderately to highly polluted (2 < I_geo_ ≤ 3) in the fine fraction (Ø3), meanwhile the finest fraction sediments (Ø4 and Ø<4) were restricted within the moderately polluted class (1 < I_geo_≤2). At Hurghada, the fine fraction sediments (Ø3, Ø4 and Ø<4) recorded remarkable pollution signs by Cd. I_geo_ of Cd was changed between unpolluted to moderately polluted bulk sediments (0 < I_geo_ ≤1) to highly polluted in Ø3 (3 < I_geo_≤4), extremely highly polluted in Ø4 fraction (4 < I_geo_ ≤5) and moderately to highly polluted (2 < I_geo_ ≤3) in Ø<4. The obtained results of I_geo_ indicated that Cd has slight signs of pollution in the bulk sediments at both locations, however noticeable evidence of pollution signs in the fine fraction sediments at Hurghada.

**Table 5:** The calculated geo-accumulation indices (*I*_*geo*_) for The different metals at The studied localities.

Locality	Sed. size	Cd	Pb	Ni	Cu	Zn	Mn	Fe
Mersa Alam	Bulk	0.65	−1.16	−0.67	−2.05	−1.61	0.97	−1.57
	Ø_3_	2.54	−0.71	−0.58	−2.46	−1.81	−2.33	−1.54
	Ø_4_	1.68	−0.99	−0.85	−2.88	−1.94	−2.56	−1.62
	Ø_<4_	1.79	−0.82	−1.06	−2.54	−1.93	−2.85	−1.97
Hurghada	Bulk	0.80	−0.39	−2.49	−3.98	−3.40	−6.13	−7.04
	Ø_3_	3.68	0.16	−2.42	−4.29	−3.76	−6.12	−6.81
	Ø_4_	4.18	0.06	−2.44	−4.71	−3.66	−5.66	−6.52
	Ø_<4_	2.93	−0.06	−2.54	−4.79	−3.65	−5.38	−6.18

**Fig. 11:**
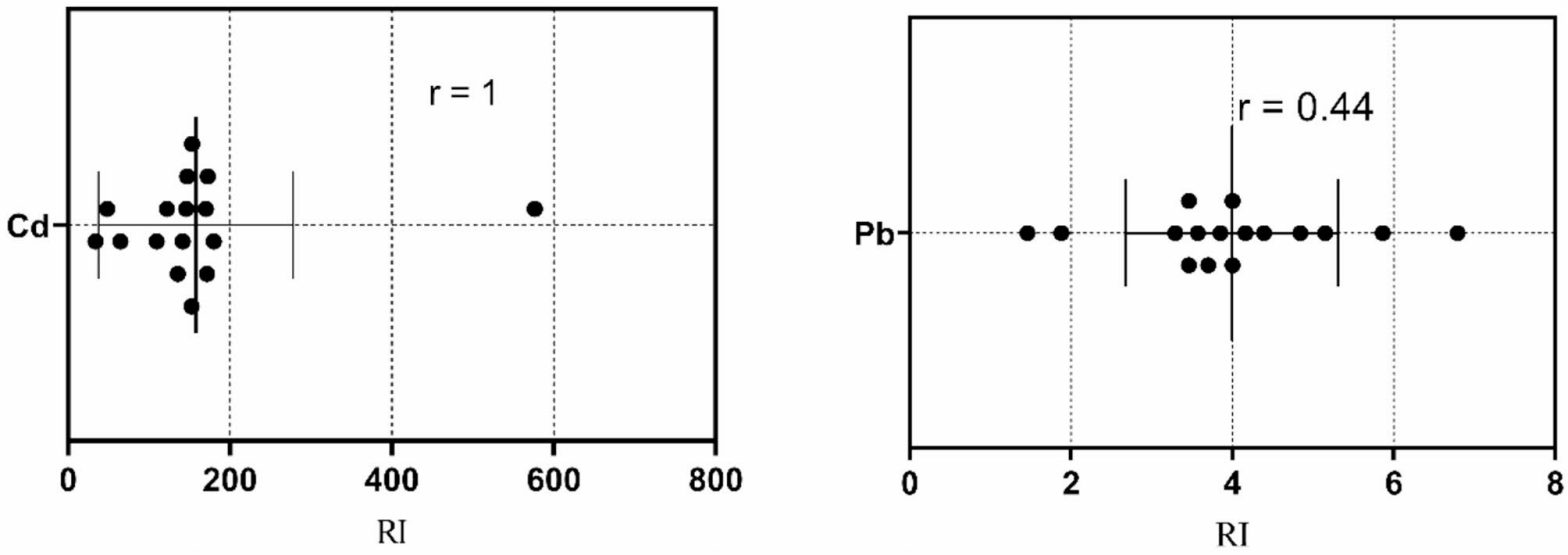
Relationship between individual ecological risk (Eri) and overall risk index (RI) for Cd and Pb at Mersa Alam.

**Fig. 12:**
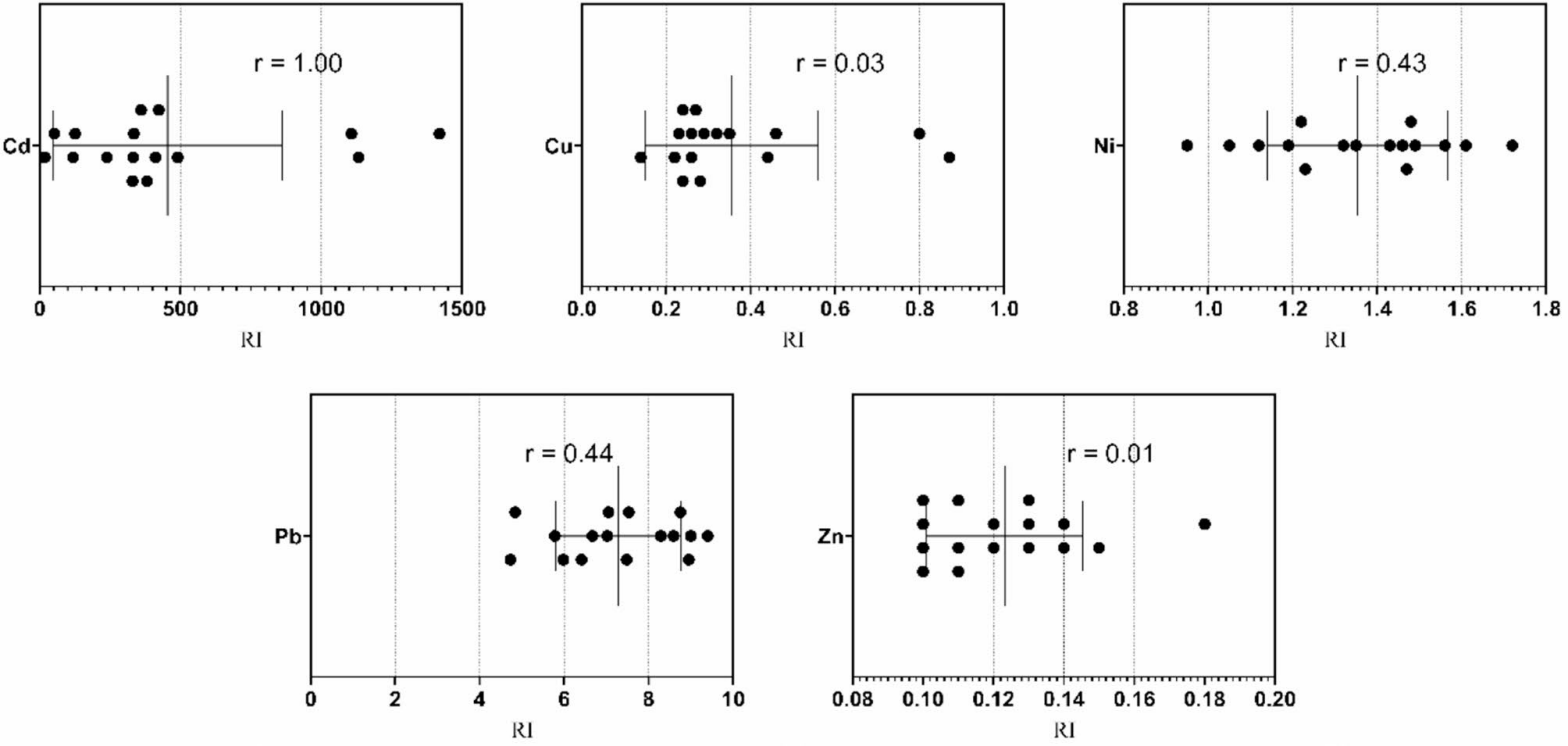
Relationship between Eri and RI for Cd, Cu, Ni, Pb, and Zn at Hurghada, highlighting their respective risk contributions.

According to the degree of contamination (C_deg_) scale, Marsa Alam showed slight pollution signs by Cd increased from moderate contamination (1–3) in the bulk sediments to very high contamination (> 6) in Ø3 (Table 6). The degree of Cd contamination (C_deg_) at Hurghada was more pronounced in the fine fractions (Ø3, Ø4, Ø<4) than in the bulk sediments indicating the anthropogenic sources of Cd (Table 6). Relative to the fine fraction percentages at each site of the studied localities, the pollution signs do not pose hazardous threats for marine organisms or humans. This investigation was supported by the individual ecological risk (Eri) and the potential ecological risk index (RI), whereas, the examined metals except Cd recorded low individual ecological risk (Eri < 40) at both localities. The calculated Eri of Cd at Marsa Alam was classified between moderate risk (40 ≤ Eri < 80) in the bulk sediments, considerable (80 ≤ Eri < 160) in Ø4 and Ø<4 fractions, and high risk (160 ≤ Eri < 320) in Ø3. The potential ecological risk index (RI) showed low risk (RI < 150) in the bulk and moderate risk (150 ≤ RI ≤ 300) in the fine fraction sediments (Table 7). However, the calculated Eri of Cd at Hurghada showed a variation between low risk (RI < 150) in the bulk and very high contamination (Eri > 320) in the different fine fractions confirming, the anthropogenic Cd pollution in the locality. The relationships between individual ecological risk (Eri) and potential ecological risk (RI) for the different metals revealed that Cd was the primary cause of the observed contamination (*r* = 1.00) and possible risk from Pb (*r* = 0.43) at Marsa Alam Fig. 11). At Hurghada, in addition to Cd (*r* = 1.00); Pb and Ni were considered a possible risk source (*r* = 0.44 and 0.43) (Fig. 12).

**Table 6:** The degree of contamination (C_deg_) for The studied metals at The studied localities.

Locality	Sed. Size	Cd	Pb	Ni	Cu	Zn	Mn	Fe
Mersa Alam	Bulk	2.35	0.67	0.95	0.36	0.49	0.33	0.51
	Ø_3_	8.71	0.92	1.00	0.27	0.43	0.30	0.52
	Ø_4_	4.80	0.76	0.83	0.10	0.04	0.25	0.49
	Ø_<4_	5.17	0.85	0.72	0.12	0.04	0.21	0.38
Hurghada	Bulk	2.62	1.15	0.27	0.05	0.02	0.02	0.01
	Ø_3_	19.27	1.67	0.28	0.08	0.11	0.02	0.01
	Ø_4_	27.27	1.57	0.28	0.06	0.12	0.03	0.02
	Ø_<4_	11.45	1.44	0.26	0.05	0.12	0.04	0.02

**Table 7:** The calculated individual ecological risk (Eri) for the different metals and the potential ecological risk index (RI) at the studied areas.

Locality	Sed. size	Cd	Pb	Ni	Cu	Zn	RI
Mersa Alam	Bulk	70.60	3.36	4.73	1.81	0.49	80.98
	Ø_3_	261.36	4.58	5.01	1.36	0.43	272.74
	Ø_4_	143.95	3.79	4.15	1.02	0.39	153.30
	Ø_<4_	155.21	4.24	3.59	1.29	0.39	164.73
Hurghada	Bulk	78.60	5.73	1.34	0.48	0.14	86.29
	Ø_3_	578.01	8.37	1.40	0.38	0.11	588.28
	Ø_4_	818.03	7.83	1.38	0.29	0.12	827.64
	Ø_<4_	343.59	7.20	1.29	0.27	0.12	352.47

### Carcinogenic risk (ILCR) probability

Some heavy metals such as; Cd, Pb, Ni, and excess Cu has been linked to an increased probability of different types of human cancer throughout the long term of exposure^51^^52^. demonstrated that the ecological risk and the potential ecological risk factor are posed by the contamination of specific metals; Cd, Pb, and Ni. According to^53^, for carcinogens with one carcinogenic element and those with several carcinogenic elements, the permissibility limits of ILCR probability are < 1 × 10 − 4 and 1 × 10^− 6^ respectively. As shown in (Table 8), the calculated data showed that the chances of cancer risk (ILCR) due to consuming the sea products from the investigated areas were improbable for Cd, Pb, and Ni throughout ingestion or inhalation. The calculated ILCR values at the studied sites were less than (< 1 × 10^− 4^) for one metal and less than (1 × 10^− 6^) for all the studied carcinogenic metals.

**Table 8:** The calculated carcinogenic risk (ILCR) for the different metals at the studied areas.

Locality	Sed. size	Ingestion			Inhalation			Total cancer risk
		Cd	Pb	Ni	Cd	Pb	Ni	
Mersa Alam	Bulk	1.6E-07	6.7E-08	6.4E-05	4.0E-10	5.1E-11	1.1E-08	6.4E-05
	Ø_3_	5.8E-07	9.1E-08	6.8E-05	1.5E-09	6.9E-11	1.0E-08	6.9E-05
	Ø_4_	3.2E-07	7.6E-08	5.6E-05	8.2E-10	5.7E-11	8.4E-09	5.7E-05
	Ø_<4_	3.5E-07	8.5E-08	4.9E-05	8.8E-10	6.4E-11	7.7E-09	4.9E-05
Hurghada	Bulk	1.8E-07	1.1E-07	1.8E-05	4.5E-10	8.7E-11	9.5E-09	1.8E-05
	Ø_3_	1.3E-06	1.7E-07	1.9E-05	3.3E-09	1.3E-10	8.2E-09	2.1E-05
	Ø_4_	1.8E-06	1.6E-07	1.9E-05	4.7E-09	1.2E-10	7.7E-09	2.1E-05
	Ø_<4_	7.7E-07	1.4E-07	1.7E-05	2.0E-09	1.1E-10	6.9E-09	1.8E-05

## Conclusions

The investigated sites had heterogeneous sediments of; lithogenous, biogenous, and authigenic origins due to the terrigenous inputs, biological productions, and the geochemical changes. These sites were highly affected by slack conditions and the slightly limited water exchange that led to continuous changes between oxic and anoxic conditions. Significant variation was observed between the two localities (Hurghada and Marsa Alam) in the carbonates production (CO_3_%) and the other sources of terrestrial inputs (natural and/or anthropogenic), which led to significant variation in the heavy metal’s concentrations. According to the findings of the investigations, Fe, Mn, Zn, Cu, and Ni were primarily obtained from the same terrestrial sources. They also have similarities in their origin and patterns of accumulation, and they have worked together in the same geochemical forms.

The obtained investigations revealed that Fe, Mn, Zn, Cu, and Ni were derived essentially from the same terrestrial sources, whereas they are similar in nature and accumulation patterns, and have been associated in the same geochemical forms much more the association with carbonate lattices. The recorded values of Cd and pb were higher at Hurghada than at Marsa Alam attributed to many anthropogenic sources. Correlation coefficient, PCA, and Geo-accumulation (Igeo confirmed the geochemical tendencies of the studied heavy metals toward Fe as oxides more than the other geochemical forms affected by the local oceanographic conditions. Also, the contamination factor (Cf), the degree of Contamination (Cdeg), the individual ecological risk (Eri), and the potential ecological risk index (RI) indicated that the pollution levels do not pose significant ecological or human health threats. In addition, the computed Carcinogenic Risk (ILCR) probability data showed the unlikely risk of inhaling or ingesting Cd, Pb, and Ni. We advise enforcing legal regulations on marine and coastal land base operations to prevent future shortages in the community structure within these areas.

## Data Availability

The datasets used and/or analysed during the current study available from the corresponding author on reasonable request.
